# Evaluation of the cost of cervical cancer at the National Institute of Oncology, Rabat

**DOI:** 10.11604/pamj.2016.23.209.7750

**Published:** 2016-04-20

**Authors:** Amine Cheikh, Sanaa El Majjaoui, Nabil Ismaili, Zakia Cheikh, Jamal Bouajaj, Chakib Nejjari, Amine El Hassani, Yahya Cherrah, Noureddine Benjaafar

**Affiliations:** 1 Team of Pharmacoepidemiology and Pharmacoeconomics, Faculty of Medicine and Pharmacy, University Mohammed V, Rabat, Morocco; 2 Department of pharmacy, International Hospital Cheikh Zaid, Rabat, Morocco; 3 Faculty of Medicine, Abulcasis University, Rabat, Morocco; 4 Department of Radiotherapy, National Institute of Oncology, Rabat, Morocco; 5 Faculty of Medicine and Pharmacy, Mohammed V University, Rabat, Morocco; 6 Department of oncology, Regional Cancer Centre, Marrakech, Morocco; 7 Department of Radiotherapy, International Hospital Cheikh Zaid, Rabat, Morocco; 8 National Institute of Public Health, Rabat, Morocco; 9 Department of Epidemiology, Faculty of Medicine and Pharmacy, Hassan II University, Fes, Morocco; 10 Head of Hospital, International Hospital Cheikh Zaid, Rabat, Morocco

**Keywords:** Cervical cancer, cost evaluation, direct cost

## Abstract

**Introduction:**

The Cervical Cancer (CC) is one of the heavy and costly diseases for the population and the health system. We want to know through this study, the first in Morocco, the annual cost of the treatment of this disease at the National Institute of Oncology (NIO) in Rabat, we also want to explore the possibility of flat-rate management of this disease in order to standardize medical practices and improve reimbursement by health insurance funds.

**Methods:**

550 patients were treated for their cervical cancer in the Rabat's NIO. Data of all of medical and surgical services offered to patients were collected from the NIO registry. The cost of care was assessed using the method of micro-costing. We will focus to the total direct cost of all the services lavished to patients in NIO.

**Results:**

The global cost was about US$ 1,429,673 with an average estimated at US$ 2,599 ± US$ 839. Radiotherapy accounts for 55% of total costs, followed by brachytherapy (27%) and surgery (7%). This three services plus chemotherapy influence the overall cost of care (p <0.001). Other services (radiology, laboratory tests and consultations) represent only 10%. The overall cost is influenced by the stage of the disease, this cost decreased significantly evolving in the stage of CC (p <0.001).

**Conclusion:**

The standardization of medical practices is essential to the equity and efficiency in access to care. The flat-rate or lump sum by stage of disease is possible and interesting for standardizing medical practices and improving the services of the health insurance plan.

## Introduction

Cervical Cancer (CC) is one of the important public health problems. It is estimated that over a million women worldwide currently have cervical cancer. Most of these women are not diagnosed, and they have no access to treatment that could cure them or prolong their survival [1]. In women, CC, the second most common cancer in developing regions (452,000 cases), is only the 10th most common cancer in developed regions (76,000 cases) [2]. Cervical cancer is the fourth most common cancer in women, and the seventh overall, with an estimated 528,000 new cases in 2012. As with liver cancer, a large majority (around 85%) of the global burden occurs in the less developed regions, where it accounts for almost 12% of all female cancers. There were an estimated 266,000 deaths from cervical cancer worldwide in 2012, accounting for 7.5% of all female cancer deaths. Almost nine out of ten (87%) cervical cancer deaths occur in the less developed regions [3]. The 14 million estimated new cases of cancer worldwide annually inflict a crushing burden of economic costs and human suffering [4, 5]. The estimated total annual economic cost of cancer was approximately US$ 1.16 trillion in 2010, the equivalent of more than 2% of total global gross domestic product [6]. Between 2.4 million and 3.7 million avoidable deaths per year, 80% of which occur in low and middle-income countries [6]. A reasonable estimate shows that the world could have saved between US$ 100 billion and US$ 200 billion in 2010 by investing in prevention, early detection, and effective treatment of cancer [6]. A 90% reduction in cases through prevention implies a reduction of at least 20% in the total estimated costs of treating cancer or approximately US$ 65 billion per year [7]. CC is by far the most common Human Papillomavirus (HPV)-related disease [8]. The number of new genital HPV infections worldwide is estimated at 30 million per year. It is estimated that 50 to 75% of women aged 15 to 44 are or have been exposed to HPV [9, 10]. Because the vaccines do not protect against all HPV types that can cause cervical cancer, girls vaccinated against HPV will still require CC screening later in their lives [1]. But in developing countries, limited access to effective screening explains that the disease is often diagnosed at a more advanced stage. The high mortality rate from cervical cancer globally (52%) could be reduced by effective screening and treatment programs [8]. The most cost-effective strategies were those that required the fewest visits, resulting in improved follow-up testing and treatment. Screening women once in their lifetime, at the age of 35 years, with a one-visit or two-visit screening strategy involving visual inspection of the cervix with acetic acid or DNA testing for HPV in cervical cell samples, reduced the lifetime risk of cancer by approximately 25 to 36%, and cost less than $500 per year of life saved. Relative cancer risk declined by an additional 40% with two screenings (at 35 and 40 years of age), resulting in a cost per year of life saved that was less than each country's per capita gross domestic product a very cost-effective result [11]. In Morocco, the Rabat cancer registry shows that 2,473 new cases of cancer were registered among residents in this city during the period 2006-2008. The overall world age-standardized rate (ASR) for all sites combined was 136.6/100,000 for men and 114.5/100,000 for women. In females, the most frequently reported malignancies were breast cancer (39.9%), followed by CC (11.4%), colorectal cancer (7.5%), Non-Hodgkin Lymphoma (NHL) (3.4%), and thyroid cancer (3.4%) [12]. We want to study, through this work, the annual cost of the treatment of this disease at the National Institute of Oncology (NIO) in Rabat. We also want to explore the possibility of flat-rate management of this disease in order to standardize medical practices and improve reimbursement by health insurance funds.

## Methods

550 patients followed retrospectively for their CC in the Rabat's NIO for one year, between first January and 31 December 2006, were included in this study. The analyzed data were extracted from the register of the Epidemiology Unit of Rabat's NIO. Patients received various services: surgery, radiotherapy, brachytherapy and chemotherapy. They have also undergone biological analyzes, anatomopathology analysis and radiological examinations. All these services, summarized in Table 1 [13], will be integrated into the analysis of the cost of the CC. Data of all medical and surgical services offered to patients were collected from the NIO registry. The unit and overall cost of care was assessed using the method of micro-costing. We will focus to the total direct cost of all the services lavished to patients in the NIO in Rabat. The calculation of the cost of services provided in this study was done between November 2012 and November 2013. The cost was measured in two ways: •The overall cost of care based on the relative costs of each service, •The overall cost based on the lump sum of the system of mandatory health insurance established in the agreements signed between the health insurance funds and the University Hospitals. Statistical analysis: Statistical analyses were performed using SPSS 17.0 for Microsoft Windows XP.

**Table 1:** Summary of services lavished on 550 patients with CC

Services	Number	Percentage (%)
**Biological analyzes**		
Blood count	428	66.21
Renal function analysis (Urea, Creatinine, Creatinine Clearance)	420	65
**Function explorations**		
Thoracic radiography	480	87.3
abdominopelvic ultrasound	44	8
Abdominopelvic CT	501	91
MRI	17	3
**Colposcopy and biopsy**		
Biopsy	550	100
**Surgery**		
Total Colpohysterectomy plus lymphadenectomy	96	17.4
Total Colpohysterectomy without lymphadenectomy	19	3.4
Total hysterectomy interadnexal	1	0.2
Colpectomy	2	0.4
NP	1	0.2
**Radiotherapy**		
Pelvis	505	92
Pelvis and Para-aortic lymphadenopathy	10	1.80
Brachytherapy		
Utero-vaginal	395	72
Low Dose Rate	334	61
High Dise Rate	61	11
**Chemotherapy**		
Concomitant	422	76.90
Neoadjuvant	21	3.80
Adjuvant	2	0.40
Palliative	7	1.30

## Results

The average age of patients was 50.72 ± 11.76 years with a predominance of the age group between 45 and 54 years (32%) followed by those between 35 and 44 years (26%) and those between 55 and 64 years (21%). The most common stage in our series is the IIb stage (37.6%) followed by IIIb (36%). IVa and IVb stages represent a very small proportion in our series (2.5%). Regional cancer is the most type of cervical cancer in our series (410 patients, 74.5%) followed by local cancer (126 patients, 22.9%) and distant cancer (14 patients, 2.5%). Localized cancer denotes the classification of invasive cervical cancer as stage Ia1, Ia2, Ib1, Ib2, or IIa, regional locally advanced as stage IIb, IIIa, or IIIb, and distant as stage IVa or IVb. The total cost of treatment of 550 patients has been estimated at $1,429,673 with an average estimated at US$ 2,599 ± US$ 839 (1$ US = 9 Moroccan dirham). Radiotherapy alone accounts for 53% of total cost, followed by brachytherapy (27%) and surgery (14%). The three services represent 94% of the overall cost of care of the CC at Rabat's NIO. Other services (chemotherapy, radiological examinations, laboratory tests, consultations) represent only the remaining 6%. The overall cost of managing patients depends significantly on the stage of the CC (Table 2), the cost decreases with the stage of the disease; (r = -0.491, p <0.001). This is mainly due to the limitation of the therapeutic choices for advanced cases (distant). In addition, there is a significant correlation between age and cancer stage (p = 0.002). This could be explained by the late diagnosis and late management in some women. On the other hand, the overall cost depends mainly for four services, radiotherapy, brachytherapy, surgery and chemotherapy (p <0.001). These services must be taken into account when setting the flat-rate of managing patients by CC stage. For the rest of the services, that is to say, radiology, biological tests, anatomopathology and others, they do not influence the overall cost, so it can be standardized in all patients at any stage or regardless of the payer.

**Table 2:** Average, minimum and maximum cost of CC by type of cancer

Stage	N	Average cost	Standard deviation	Minimum	Maximum
Local	126	3,093.9	1,215.2	934.4	5,938.0
Regional	410	2,473.8	614.2	934.4	5,652.4
Distant	14	1,776.3	107.8	1,608.0	1,951.4

## Discussion

We have analyzed a large number of published studies on the cost of CC, few have addressed the cost of treatment and management of patients and most have focused on the cost-effectiveness of diagnostic or vaccination but there are fewer published studies that assess screening and vaccination in developing countries. M. E. van den Akker-van Marle et al have demonstrated through the analysis of 500 screening policies that there were 15 efficient screening policies. For the efficient policies, the predicted gain in life expectancy ranged from 11.6 to 32.4 days, compared with a gain of 46 days if cervical cancer mortality were eliminated entirely. The average cost-effectiveness ratios increased from US$ 6,700 (for the longest screening interval) to US$ 23,900 per life-year gained. They concluded that the basis for the diversity in the screening policies among high-income countries does not appear to relate to the screening policies’ cost-effectiveness ratios, which are highly sensitive to the number of Pap smears offered during a lifetime [14]. Van Rosmalen et al have demonstrated that under the base-case assumptions, primary HPV testing with cytology triage is the most cost effective strategy. Using cost-effectiveness thresholds of US€ 20,000 and US€ 50,000 per Quality-Adjusted Life-Year (QALY) gained yields optimal screening programs with three and seven screening rounds, respectively [15]. On the evaluation of the cost-effectiveness of the vaccine against HPV, Jane J. Kim et al have demonstrated that the cost-effectiveness ratio of vaccination of 12-year-old girls in USA was US$ 43,600 per QALY gained, as compared with the current screening practice. Under baseline assumptions, the cost-effectiveness ratio for extending a temporary catch-up program for girls to 18 years of age was US$ 97,300 per QALY; the cost of extending vaccination of girls and women to the age of 21 years was US$ 120,400 per QALY, and the cost for extension to the age of 26 years was US$ 152,700 per QALY [16]. They concluded that the cost-effectiveness of HPV vaccination will depend on the duration of vaccine immunity and will be optimized by achieving high coverage in preadolescent girls, targeting initial catch-up efforts to women up to 18 or 21 years of age, and revising screening policies [16]. In Canada, Anonychuk et al found that vaccination was estimated to cost $18,672-$31,687 per QALY-gained, the lower range representing inclusion of cross protective efficacy and herd immunity [17]. They concluded that in the context of current screening patterns, vaccination of 12-year old Canadian females with a cervical cancer vaccine is estimated to significantly reduce cervical cancer and mortality, and is a cost-effective option. However, the economic attractiveness of vaccination is impacted by the vaccine′s duration of protection and the discount rate used in the analysis [17]. In our context, the importance of assessing the overall cost of the treatment of cancer in developing countries is to assess the overall budget for the monitoring and treatment of patients in accordance with international standards. Once the budget is known, the hospital asked the financial resources of the ministry of health or NGOs that allow it to provide the necessary patient care (radiotherapy, brachytherapy, surgery and chemotherapy) while ensuring efficiency and equity in access to care.

In addition, the flat-rate or sum lump has the advantage of offering patients who are classified in the same stage of disease the same quality care regardless of their health insurance plan or socio-economic level. This is, unfortunately, not always guaranteed in countries with limited economic means. In our case, the fact that radiation is the most significant burden could be explained by important elements: cost or package radiotherapy representing on average 50% of total costs and the frequency of this act in our series (94%). The same observation could be registered for brachytherapy but its frequency was lower than radiotherapy. As for the surgery, and despite the high cost of the latter, its frequency was low in our series compared to the two previous services, it was made for only 23% of patients followed. The cost of care is affected by stages of cancer. Referring to the results of our study, the standard deviations from the average costs are lower for stage IVA and IVB. In the early stages, ie IB1, IIA, IIB and III, standard deviations are significant and important work to standardize medical practices should be undertaken within the NIO. In addition, the low cost of anticancer drugs (cisplatin and fluoro-uracil) and chemotherapy protocols proposed in the management of the CC at NIO has significantly reduced the impact of this act on the overall cost of care. Otherwise, the flat-rate to manage CC will no doubt bring substantial benefits to the hospital structure:

**Standardization of medical practices:** As concluded in our work, a package could be applied by stage of CC. In this case, care providers will be encouraged to follow the protocols set up by the working groups without a concern about the overall amount of support or funding capacity of patients. The most important is that patients benefit from optimal management according to current guidelines (Table 3).

**Table 3:** Minimum and maximum cost of CC by type of cancer (based on the international standards)

Stage	Minimum	Maximum
Local	1 218.9	5 270.2
Regional.	5 270.2	6 981.3
Distant	552.2	6 981.3

Management of information's flow and patients: an integrated information system, or lack of it a system of record management and medical records would be an asset and an advantage of the flat-rate of support CC. The mastery of medical, administrative and financial information are easier with fixed flat-rate instead of adopting a plethora of database according to the stages of the disease, the social level of the patient and the health insurance funds which they are affiliated. Once the flat-rate is fixed between the health insurance funds and hospitals, insurance funds could pay the hospital depending on the stage of the cancer (local, regional or distant) and the number of patients. We made an evaluation of the overall cost of care based on international standards. The result of this work is summarized in the table below: Health insurance plan: Reimbursement of a flat-rate or a lump sum has advantages but also can cause loss of information on services really provided by health care providers or hospitals. However, and taking into account, on the one hand, the large flow records management and reimbursement that flood the health insurance funds, and secondly the lack of staff processing and controlling of these records, the flat-rate support for the CC stage could be a solution to improve fund performance and creditworthiness of establishment of public care.

## Conclusion

The CC is one of the most common cancers in women and deadly. The management of this type of pathology firstly requires optimal access of women to prevention and early diagnosis, and later to the appropriate therapeutic measures depending on the stage of disease and socioeconomic level the patient. In developing countries where about 90% of all cervical cancer deaths occur, we assist to a public health failure because infrastructure and technological expertise for cytology programs are not available and three visits for screening, diagnosis, and treatment are not feasible. For countries with limited resources, screening efforts should target women age 35 or older, and efforts should focus on attaining high coverage of single lifetime screening before increasing the frequency of screening.

### What is known about this topic

- CC is one of the important public health problems (It is the fourth most common cancer in women and the seventh overall with an estimated 528,000 new cases in 2012);
- The cost of care of cervical cancer is very important, and many middle-income countries cannot cover the needs of all women who suffer from this cancer;
- Early detection of cancer in the early stages can prevent many deaths and make big savings.

### What this study adds

- The results of this study demonstrate that the cost of care is affected by stages of cancer ie the cost decreases with the stage of the disease. This is mainly due to the limitation of the therapeutic choices for advanced cases (distant);
- Referring to the results of our study, the standard deviations from the average costs are lower for stage IVA and IVB but in the early stages, ie IB1, IIA, IIB and III, standard deviations are significant and important work to standardize medical practices should be undertaken within the NIO;
- When a sum lump can be applied by stage of CC, care providers will be encouraged to follow the protocols set up by the working groups without a concern about the overall amount of support or funding capacity of patients. In this case, the most important is that patients benefit from optimal management according to current guidelines.
